# AI in breast screening mammography: breast screening readers' perspectives

**DOI:** 10.1186/s13244-022-01322-4

**Published:** 2022-12-09

**Authors:** Clarisse Florence de Vries, Samantha J. Colosimo, Moragh Boyle, Gerald Lip, Lesley A. Anderson, Roger T. Staff, the iCAIRD Radiology Collaboration, D. Harrison, C. Black, A. Murray, K. Wilde, J. D. Blackwood, C. Butterly, J. Zurowski, J. Eilbeck, C. McSkimming

**Affiliations:** 1 Aberdeen Centre for Health Data Science, Institute of Applied Health Sciences, University of Aberdeen, Aberdeen, Scotland; 2 Aberdeen Royal Infirmary, National Health Service Grampian (NHSG), Aberdeen, Scotland; 3 School of Medicine, Medical Science and Nutrition, University of Aberdeen, Aberdeen, Scotland

**Keywords:** Mammography, Screening, Survey, Radiologist, Breast screening reader

## Abstract

**Objectives:**

This study surveyed the views of breast screening readers in the UK on how to incorporate Artificial Intelligence (AI) technology into breast screening mammography.

**Methods:**

An online questionnaire was circulated to the UK breast screening readers. Questions included their degree of approval of four AI implementation scenarios: AI as triage, AI as a companion reader/reader aid, AI replacing one of the initial two readers, and AI replacing all readers. They were also asked to rank five AI representation options (discrete opinion; mammographic scoring; percentage score with 100% indicating malignancy; region of suspicion; heat map) and indicate which evidence they considered necessary to support the implementation of AI into their practice among six options offered.

**Results:**

The survey had 87 nationally accredited respondents across the UK; 73 completed the survey in full. Respondents approved of AI replacing one of the initial two human readers and objected to AI replacing all human readers. Participants were divided on AI as triage and AI as a reader companion. A region of suspicion superimposed on the image was the preferred AI representation option. Most screen readers considered national guidelines (77%), studies using a nationally representative dataset (65%) and independent prospective studies (60%) as essential evidence. Participants’ free-text comments highlighted concerns and the need for additional validation.

**Conclusions:**

Overall, screen readers supported the introduction of AI as a partial replacement of human readers and preferred a graphical indication of the suspected tumour area, with further evidence and national guidelines considered crucial prior to implementation.

**Supplementary Information:**

The online version contains supplementary material available at 10.1186/s13244-022-01322-4.

## Key points

1. Surveyed UK breast screening readers supported the introduction of AI.
2. Respondents approved of the replacement of one of the two initial readers.
3. Participants objected to the replacement of all human readers.
4. Respondents preferred the AI to graphically indicate the suspected tumour area.
5. Screen readers preferred forms of evidence based on national guidelines, national representative datasets, and independent prospective studies.

**Supplementary Information:**

The online version contains supplementary material available at 10.1186/s13244-022-01322-4.

## Introduction

Artificial intelligence (AI) has the potential to transform medical care. Early optimism on the potential use of AI in radiology led to the viewpoint that the replacement of radiologists was imminent [1, 2]. However, more recent views, citing the history of automation and the complex responsibilities of the radiologist that expand beyond image interpretation, have suggested that radiologists’ jobs will evolve rather than disappear [2–4]. Recent surveys have shown that most radiologists are favourable to the adoption of AI in clinical practice [7–9]. However, despite a willingness to implement AI, there are significant barriers to realising the potential clinical and operational gains. A demonstration of efficacy, robustness and safety is needed. The incorporation of AI technology into breast screening has received considerable attention and investment [5, 6]. A review of 23 studies on AI for breast cancer screening (2010–2018) found that most were small, retrospective studies using cancer-enriched datasets and did not include a real-world external validation [10]. A 2021 systematic review concluded that currently there is insufficient evidence to support the implementation of AI in breast cancer screening [5].

Furthermore, there is no consensus on what types of evidence would be considered sufficient to implement an AI breast screening tool into the screening pathway. Radiologists expect breast imaging to be among the radiology subspecialties most likely to be influenced by AI technology [9]. However, prior surveys did not assess the views of radiologists/screen readers in a mammography screening setting. Breast screening readers are highly specialised roles requiring certification, a minimum annual read of 5000 screening mammograms and participation in quality assurance activities [11]. This professional group will be most directly affected by mammography AI.

The opinion of the professional groups directly affected by AI is essential to carry out efficient practical developments in the clinic. This study is the first survey of the UK breast screening readers’ attitudes towards the implementation of AI in the breast screening service. Mammographic readers were surveyed for their views on how to implement AI in clinical practice and the types of evidence deemed necessary to introduce AI into their workplace.

## Methods

### Questionnaire design

The National Health Service in the UK offers publicly funded breast screening to all women between 50 and 70 every 3 years. Two expert readers interpret each mammogram, with disagreements resolved by a third reader (arbitration). In May 2020, we sought to obtain the views of the mammographic screening community on AI in interpreting breast screening mammograms using a standardised online questionnaire. The questionnaire was validated through consultation with leading mammography readers and social scientists in the UK. Regional and national professional screening groups were approached to advertise the study. The complete questionnaire is available in the Additional file 1.

Respondents were asked to confirm that they were nationally accredited breast cancer screening readers. Information was collected about their job title, years of experience, understanding of AI and views of AI use in medical screening. Self-reported non-accredited readers were excluded.

Participants were asked to indicate their level of approval of the following four scenarios on a five-point Likert scale (Strongly Object, Object, Neutral, Approve, and Strongly Approve), and to list them in order of preference:

1. A partial replacement scenario: Instead of two specialists examining a participant’s mammograms, a specialist and an AI algorithm examine the mammograms. If they disagree, a different specialist will make the final decision.
2. A total replacement scenario: The AI algorithm examines the mammograms without input from specialists and makes the final decision.
3. A triage scenario: The AI algorithm initially examines the mammograms. If the scan is very likely to be normal, the participant would not be invited back for further investigation. If the AI findings are indeterminate or abnormal a specialist would review the image.
4. A companion scenario: All mammograms continue to be examined by specialists as is the current practice. They will have on demand access to an AI algorithm to help them make their decisions.

Readers were also asked whether the first, second or third reader/arbitration panel should have access to the AI opinion.

Next, readers were asked which evidence would convince them to introduce AI in their workplace: performance data from vendors, national guidelines, independent retrospective studies, independent prospective studies, and/or studies using a local or national dataset.

Participants were then asked whether it is their view that the second specialist is blinded to the first reader's opinion and whether it is their view that the specialist should be blinded to the AI’s opinion.

Finally, readers were asked to rank five AI representation options (discrete opinion, mammographic scoring, percentage score with 100% indicating malignancy, region of suspicion and heat map) and whether they had been involved in the procurement of similar medical software for their organisation. A free-text option was provided for comments.

### Statistical analysis

Spearman’s rank correlations coefficients were calculated between readers’ self-reported understanding of AI and their views on the use of AI in medical screening and their approval of the four AI implementation scenarios.

### Content analysis

Content analysis was performed manually on the free-text comments by dividing them into themes. Comments were grouped and described alongside the related closed-ended survey questions. Comments which were not directly relevant to any of the closed-ended questions were described separately.

## Results

The survey had 87 nationally accredited respondents; 73 (83.9%) completed the survey in full. Most (61%) had over 10 years’ experience, and 77% were consultant radiologists. Nineteen participants provided comments. While just over a third (37%) described their understanding of AI as good or excellent, 63% had a positive or strongly positive view of AI use in screening. One respondent indicated: “I am in favour of adopting AI in mammogram reporting.” Another respondent stated: “AI has a role in breast screening and would help to alert. [AI would] [a]lso help with personnel shortage.” Most (82%) had not been previously involved in procuring similar medical software for their organisation.

**Fig. 1:**
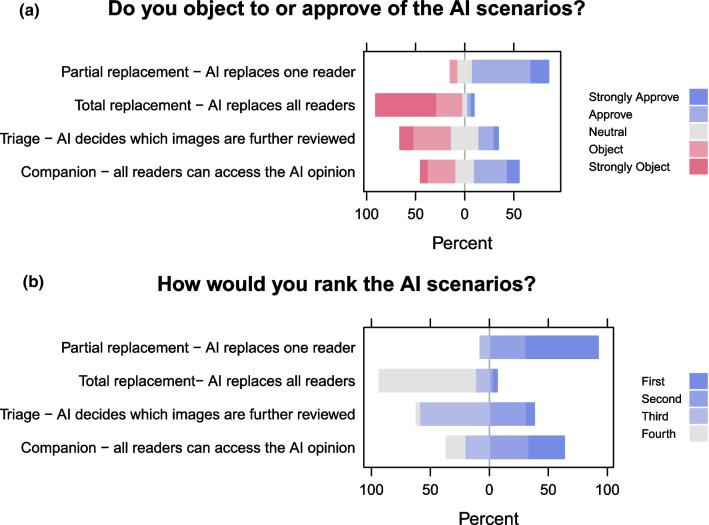
How should AI be implemented? **a** Participants were presented with four scenarios on the use of AI in breast screening and were asked to select the options that most closely reflect their views. **b** Participants were asked to rank the four AI scenarios in order of preference

Figure 1 shows participants’ responses to which AI implementation scenario they would prefer. Respondents preferred partial replacement (AI replaces one human reader) over other AI implementation scenarios. They objected to the total replacement scenario, while views on the triage and companion scenarios were mixed.

Two respondents suggested alternative AI implementation scenarios. One comment stated that “[i]t would be great to have AI tested against previous interval cancers as this is one of the few things that will influence outcomes / breast cancer mortality in the screened population” and that AI could be used “on all those cases given normal results by the readers as a safety net system prior to results being sent out.” The second response suggested that double reading with AI would not save a lot of radiology time, and that AI would be better used to maximise image quality, decide whether to perform breast imaging with tomosynthesis, pre-read symptomatic mammograms, and focus on risk and masking from breast density/parenchyma.

Approximately half of the respondents thought first readers (52%) and second readers (51%) should have access to the AI opinion. Most respondents (68%) thought that third readers or an arbitration panel should have access to the AI opinion.

**Fig. 2:**
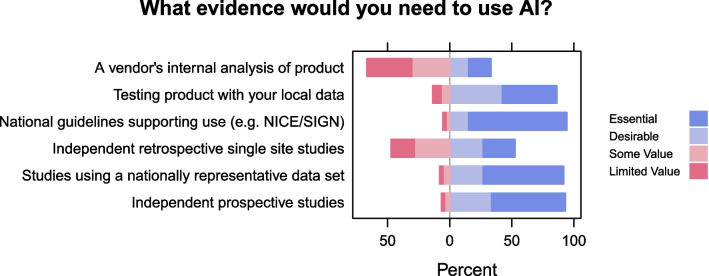
What evidence do you need to introduce AI into your workplace? Participants were asked: ‘What type of evidence would convince you of the value and utility of AI in breast screening and support AI introduction into your workplace setting?’

Figure 2 shows participants’ responses to what evidence they think would support AI introduction into their workplace. Most respondents rated national guidelines (77%), studies using a nationally representative dataset (65%) and independent prospective studies (60%) as essential to support the introduction of AI into clinical practice. Vendor generated evidence, however, was considered to have limited value. Most participants indicated that evidence generated from local data was either essential (43%) or desirable (42%).

Seven comments discussed the need for additional evidence and validation of AI breast screening tools, including different software, the threshold for recall and readers’ interactions with the AI. Related comments stated: “Replies non-committal because I want to see the evidence first!”, “I am strongly in favour of adopting AI in screening mammography reading once it has been validated and made user friendly” and “AI has so far shown excellent results with better than human sensitivity and specificity but needs input of robust data and validation tests locally and nationally.” One respondent suggested that a national working group of AI specialists and screen readers should be developed through the Royal College of Radiologists to evaluate and test the various AI systems and ways of using them on large datasets. They added: “National guidelines are vital to ensure it is used in the optimal manner and to provide medicolegal protection.”

The view that the second specialist is blinded to the first reader’s opinion was held by 45% of participants; 54% indicated that it was their view that the specialist should be blinded to the AI opinion. Two respondents indicated that they were unsure whether the question on the blinding of the second specialist to the first reader referred to whether they are currently blinded or whether they should be blinded.

**Fig. 3:**
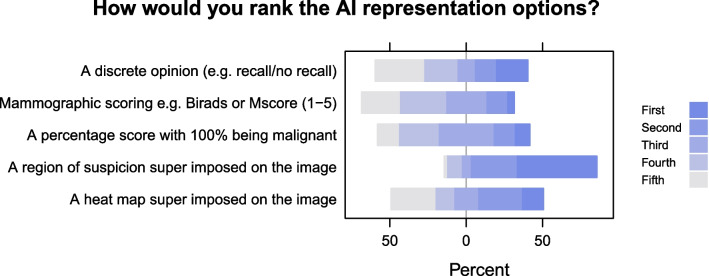
How should the AI opinion be represented? Participants were asked: “If you were able to see the AI opinion as in the companion scenario, how best do you think this should be represented?” Participants were shown five options and were asked to rank them from most to least preferred

Figure 3 shows participants’ responses to how they would rank the given AI representation options. Respondents preferred a region of suspicion superimposed on the image over other shown AI representation options.

Readers with a greater self-reported knowledge of AI were more likely to view the use of AI in medical screening as positive (*ρ* = 0.496, *p* < 0.001). Self-reported knowledge of AI was not significantly associated with approval of any of the AI implementation scenarios (*p* > 0.05).

The remaining free-text comments predominantly related to concerns regarding the introduction of AI into breast screening, including lack of planning for the needed infrastructure, and potential negative effects on screen readers, patients, and screening centres. One participant indicated that it is “[i]mportant that training of future mammographic readers is not forgotten, as AI cannot assess patients.” Relatedly, one respondent stated: “There needs to be widespread understanding of the limitations of AI as I am afraid that readers will have too much faith in its abilities.” Another participant commented: “AI will decrease specificity and increase recall rates. Radiologists will be left to cope with the fall out at assessment clinics. How can centres be assessed for QA [quality assurance] if AI is introduced?” One respondent indicated that AI is “[d]ifficult to introduce” and “buy-in from most radiologist[s]” must be obtained before introducing AI in breast screening nationally. They further stated that ethical questions should be answered in a FAQ (frequently asked questions document) to reassure screen readers. One screen reader responded: “I believe it is inevitable that AI will be introduced over the next few years and we need to ensure it is done so in the most effective manner for the breast screening programme.”

## Discussion

The survey results confirm that breast screening readers from the UK favour the introduction of AI. Those with higher self-assessed knowledge were more positive about implementing AI in breast screening. Study participants preferred the combined AI and human reader option, where AI would replace one of the initial two readers. They would also prefer the AI program to indicate the suspected tumour area graphically. Readers reported a preference for various forms of evidence: guidance from a national assessment body such as NICE, studies using a nationally representative dataset and independent prospective studies.

The main strength of this study was targeting screen readers since they are potential users of AI in the breast screening service. Our findings add to previous research which highlighted women’s views on AI for breast screening [12, 13]. Overall, women of screening age were positive towards the introduction of AI into breast screening in combination with human readers. However, a significant minority expressed negative or mixed views towards AI, with concerns including the safety of the technology and a lack of human involvement [13]. Both groups favour AI as a partial replacement over AI as a full replacement of human readers. However, while women who attended breast screening approved of AI as a companion, screen readers’ views were mixed. There are currently over 800 NHS breast screening readers in the UK [14]. Approximately 10% responded to the questionnaire, limiting the sample size. Views on whether second readers are blinded to the first are mixed. However, as this question was potentially ambiguous, it is unclear whether respondents indicated whether second readers are currently blinded or should be blinded. Across the UK, there is variation in terms of blinding of the second reader.

The path to implementation of AI technology in breast cancer screening remains unclear. The results here indicate that readers support the use of AI as a partial replacement (AI replaces one human reader) and object to AI replacing all human readers. However, most studies to date have evaluated AI breast screening algorithms as stand-alone systems and have not considered its interaction with human readers [5]. This weakness in the literature suggests that more real-world testing scenarios are required.

Most current evidence for AI in breast screening has been generated with vendor involvement and is considered to be insufficient to support its implementation [5, 15]. Readers’ broadly positive views therefore seem at odds with both their limited confidence in vendor-generated data and the available evidence. Readers may be unaware of the quality of existing evidence and potential for publication bias. However, in the free-text responses readers highlighted their concerns and need for additional testing, which suggests that their support is conditional on robust validation first taking place.

The participants indicated that they would strongly value performance data from a nationally representative dataset. Such a dataset would allow product comparison on a level playing field and enable vendors to benchmark their products. AI algorithmic bias is a concern, and its elimination is part of the US Food and Drug Administration (FDA) action plan for AI-based software as a medical device [16]. Local testing and optimisation could help reduce algorithmic bias by ensuring an AI tool works in the local setting [17]. However, not all settings will have the facility or resources to test and optimise an AI tool; a national dataset of sufficient size and diversity to mimic local settings might be a suitable alternative.

Our findings show that screen readers would like to see guidance from authorities before implementing AI into their workflow. For this to happen, however, the type, quantity, and quality of the evidence available must improve. Vendors should consult with service users and patients when designing AI technologies, as do health care providers when considering how an AI tool might be implemented into the health service.

Overall, screen readers were positive towards the introduction of AI into breast cancer screening, preferring the replacement of one of the two initial readers and a graphical indication of the suspected tumour area over other implementation options. Readers also considered additional evidence, including national guidelines, essential prior to the implementation of AI into their workplace.

## Supplementary Information

**Additional file 1.** Questionnaire and survey data (aggregated statistics).

## Data Availability

The survey data analysed during the current study (aggregated statistics) and the complete questionnaire and are available in the Additional file 1.
